# Characteristics of Allan-Herndon-Dudley Syndrome in Chinese children: Identification of two novel pathogenic variants of the *SLC16A2* gene

**DOI:** 10.3389/fped.2022.1050023

**Published:** 2022-11-15

**Authors:** Qiang Zhang, Qi Yang, Xunzhao Zhou, Zailong Qin, Shang Yi, Jingsi Luo

**Affiliations:** The Maternal and Child Health Care Hospital of Guangxi Zhuang Autonomous Region, Guangxi Birth Defects Prevention and Control Institute, Nanning, China

**Keywords:** Allan-Herndon-Dudley syndrome, *SLC16A2*, MCT8, thyroid hormone transporter, X-linked mental retardation

## Abstract

**Objective:**

The aim of this study was to identify causative variants associated with Allan-Herndon-Dudley syndrome (AHDS) in two unrelated Chinese families, and to determine their potential pathogenicity. We also summarized the core clinical symptoms of AHDS by reviewing the related literature.

**Methods:**

Genomic DNA was isolated from the peripheral blood of AHDS patients and their family members. Whole exome sequencing (WES) was performed on the proband from each family to identify the candidate variants. Subsequently, Sanger sequencing was used to verify the identified candidate variants and to assess co-segregation among the available family members. *In silico* prediction combined with 3D protein modeling was conducted to predict the functional effects of the variants on the encoded protein.

**Results:**

Two novel hemizygous variants of *SLC16A2*, c.1111_1112insGTCTTGT (Gly375fs*6) and c.942delA (Val315fs*28), were detected in two patients. We compared the clinical symptoms of the patients with all patients with AHDS reported in China and those reported in the literature. While both our patients presented symptoms mostly consistent with AHDS, Patient 1 had no abnormal brain structure and thyroid function, and yet showed other symptoms including lactic aciduria, conjunctival hyperemia, vomiting, laryngeal stridor, low immunoglobulin and iron levels.

**Conclusions:**

This study expands the mutation spectrum of AHDS and has clinical value for variant-based prenatal and postnatal screening for this condition. Doctors often have difficulty identifying AHDS by using clinical symptoms. WES can help to identify specific disorder when diagnosis cannot be made based on symptoms alone.

## Introduction

Allan-Herndon-Dudley syndrome (AHDS) was first reported by Allan in 1944 (1). In 2004, it was confirmed that AHDS is caused by variants in the *SLCI6A2* gene which is located in the Xq13.2 region of chromosome (chrX:73,641,328–73,753,751 GRCh37/hg19) (2, 3). The *SLCI6A2* gene spans 112,424 bases, contains six exons and five introns, and is translated into a protein consisting of 539 amino acids. Although the incidence of AHDS is currently unknown, the number of reported cases in the literature so far suggests that the syndrome is more common than we previously thought.

AHDS is the result of loss of function of MCT8, which is the protein product of the *SLC16A2* gene. MCT8 is an integral membrane protein that acts as a thyroid hormone transporter. It facilitates the cellular importation of T2 (diidothyronine), T3 (triiodothyronine), T4 (thyroxine) and rT3 (reverse triiodothyronine), and it plays a critical role in the development of the nervous system (4). MCT8 is also thought to play a key role in the neuronal uptake of thyroid hormone by T3 and endothelial cells allowing the hormone to cross blood-brain barrier. MCT8 deficiency can result in a decrease of T3 to nuclear T3 receptors. Therefore, the T3 reduction in brain cells may contribute to a severe neurodevelopmental deficit in males with AHDS (5, 6). Most pathogenic variants of AHDS result in either reduced activity or complete inactivation of MCT8. However, variants that result in incomplete inactivation of the protein can lead to a milder phenotype (7). Here, we report two novel frameshift variants of *SLC16A2* which were revealed by whole exome sequencing (WES) in two Chinese boys. These findings will help to further expand the mutation spectrum and provide potential novel clinical features of AHDS.

## Materials and methods

### Next generation sequencing

Both patients were examined by using WES. Genomic DNA samples were collected and sequence libraries were constructed using the Agilent Sure Select Human Whole Exome V2 Kit (Agilent Technologies, Santa Clara, CA). The prepared libraries were sequenced using the HiSeq2500 System (Illumina, San Diego, CA). Reads obtained from the BWA software package (v. 0.7.15) were mapped with the human reference genome (GRCh37/hg19). Variant calling and variant annotation were performed by using the Genome Analysis Toolkit (GATK) and further variant annotation and prioritization were performed by using the TGex software (LifeMap Sciences, Inc.v5.7).

### Sanger sequencing confirmation

A 2.5 ml of venous blood sample was taken from all the family members and Sanger sequencing was performed to confirm the presence of the variant. The following primers designed by primer3 were used: 5′-TGCTGGGGGATAAGATCAAG-3′ and 5′-GCCCACTCTGGTATTCCTCA-3′ for c.942delA (Val315fs*28) and 5′-CTCCTCTTTCTCCTCCTGTT-3′ and 5′-GCTCTCACAGAATCCTCACTCA-3′ for c.1111_1112insGTCTTGT (Gly375fs*6).

### Bioinformatic analysis and verification of observations

The bioinformatics tools SIFT (http://sift.jcvi.org/), Mutation Taster software (http://www.mutationtaster.org/), Provean (http://provean.jcvi.org/seq_submit.php), and NMDEscPredictor (https://nmdprediction.shinyapps.io/nmdescpredictor/) were used to predict the impact of the variants on protein function. The protein 3D structures of *SLC16A2* were generated by using the Swiss-Model server (https://swissmodel.expasy.org/) and the ACMG/AMP variant classification guidelines were used for variant classification (8).

## Results

### Clinical data

Proband 1 was a boy admitted to our hospital at the age of one month with no response to sound, lack of movements, poor facial expression, unsteady gaze, hypertonia, laryngeal stridor, feeding difficulties and neonatal pneumonia. The boy had a twin brother. Both were born at 37 weeks of gestation to healthy, non-consanguineous parents. He was a product of a breech birth with a weight and length of 2.68 kg and 50 cm, respectively. No neonatal asphyxia was observed during the delivery. The Apgar scores were excellent (Apgar score: 10/10/10). This boy showed poor sucking and had difficulty with swallowing. Meanwhile, he had frequent respiratory infections as an infant. His mother suffered from gestational diabetes and hypothyroidism during her pregnancy.

Physical examination of the patient's length (52 cm), weight (3.04 kg) and head circumference (33 cm) were collected. His physical examination revealed malnutrition, apathy, slow response, ankle clonus, muscle hyperirritability, neck muscle weakness, conjunctival hyperemia and laryngeal stridor. His hearing, vision and skeletal system were normal. A routine ECG showed sinus tachycardia.

**Figure 1:**
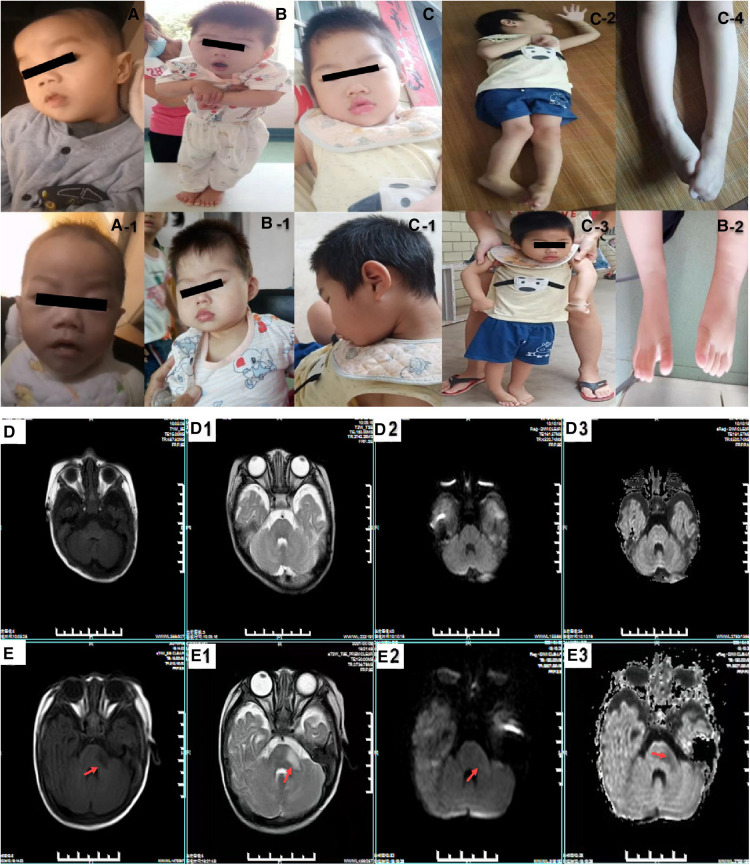
The clinical features of the probands with Allan-Herndon-Dudley Syndrome. (**A**,**A-1**) show the phenotypic characteristics of patient 1: fixed facial expression and neck muscle weakness. (**B**–**B-2**) show the phenotypic characteristics of patient 2: fixed facial expression and muscle weakness. (**C**–**C-4**) show the facial features and muscle weakness and special posture of patient 2's elder brother. (**D–D3**) show the cranial MRIs of patient 1 (normal). (**E–E3**) are patient 2 cranial MRIs. (**E,E–E3**) represent T1WI, T2WI, a high b-value DWI and ADC images, respectively. The results reveal a patchy long T1 and a long T2 signal shadow in the left mid-cerebellar peduncle, with a slightly high signal on high b-value DWI and a slightly low signal on ADC images.

Ultrasonography of the liver, reproductive system and kidneys were normal. Magnetic resonance imaging (MRI) and neonatal echocardiography were also normal (Figures 1D,D1–D3). He was treated for infection in hospital and discharged thereafter. At 5 months of age, the boy presented with worsening laryngeal stridor symptoms with sputum production and vomiting. He continued to present with failure to thrive and limb hypertonia with dull eyes, poor adaptability and gross motor development.

The main findings biochemical and metabolic tests included euthyroidism (T3: 3.15 nmol/L, normal range 1.23–4.22 nmol/L, T4: 80.97 nmol/L, normal range 69.6–219 nmol/L, free T3: 6.71 pmol/L, normal range 3.0–9.28 pmol/L, free T4: 12.19 pmol/L, normal range 11.5–28.3 pmol/L, Thyrotropin (TSH): 3.48 uIU/ml normal range 0.72–11 uIU/ml*)*; elevated white blood cell (12.80*10^9^/L, normal range 4.00–10.00*10^9^/L), lymphocyte percentage (149.00%, normal range 20%–40%) and lactic acid (4.50 mmol/L, normal range 0.50–1.60 mmol/L) and reduced iron level (9.00 μmol/L, normal range 11–30 μmol/L). The total serum protein was 58.00 g/L (normal range 60.00–80.00 g/L), albumin 42.30 g/L (normal range 35.00–55.00 g/L), immunoglobulin G 2.62 g/L (normal range 7.00–16.00 g/L), immunoglobulin A 0.18 g/L (normal range 0.70–3.30 g/L), and immunoglobulin M 0.38 g/L (normal range 0.50–2.20 g/L). His twin brother had similar symptoms and his elder sister had a normal phenotype.

**Figure 2:**
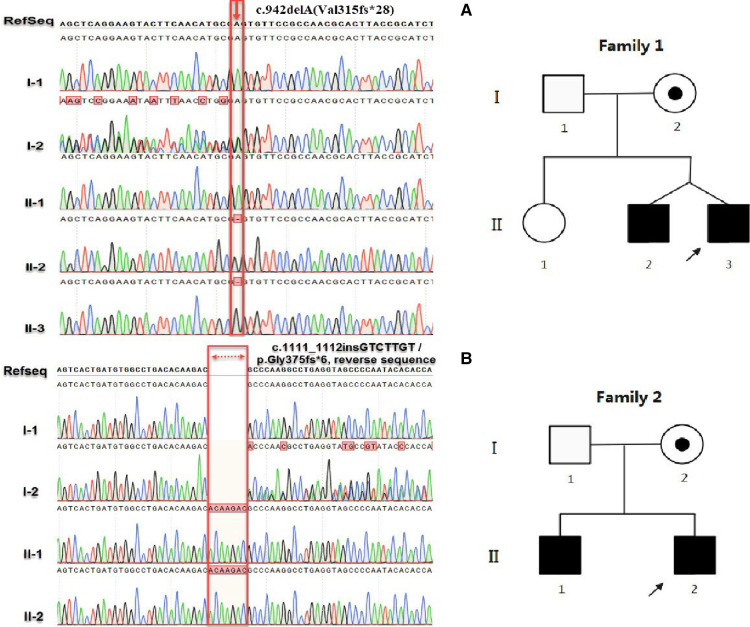
(**A**) represents the sequencing results of c.942delA(Val315fs*28) and (**B**) is the sequencing results of c.1111_1112insGTCTTGT (Gly375fs*6). In family 1, I-1, I-2, II-1, II-2 and II-3 represent the father, mother, elder sister, elder brother and the proband, respectively. In family 2, I-1, I-2, II-1 and II-2 represent the father, mother, elder brother and the proband, respectively. In the pedigree, the filled and open symbols represent the affected and unaffected individuals, respectively; A dot inside a circle represents the carriers; The squares and circles represent the males and females, respectively; The arrows indicate the probands.

**Figure 3:**
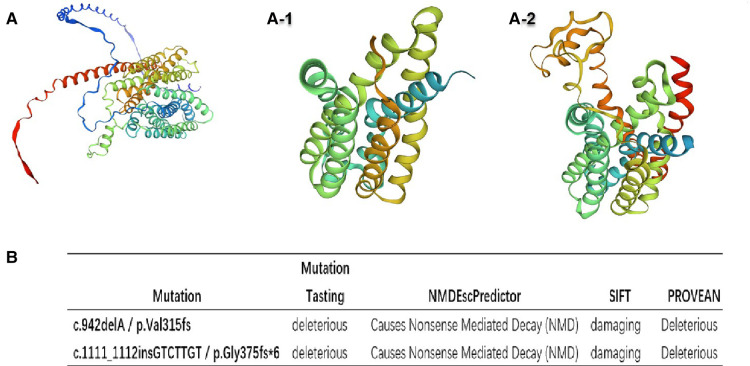
The three-dimensional structure modeling predicts a decrease in protein length with (**A**) as the wild-type and (**A-1**) and (**A-2**) as the mutant-types (**A-1**: p. Val315fs*28; A2: p. Gly375fs*6), respectively. (**B**) The effects of these variants were predicted using four different software packages.

**Figure 4:**
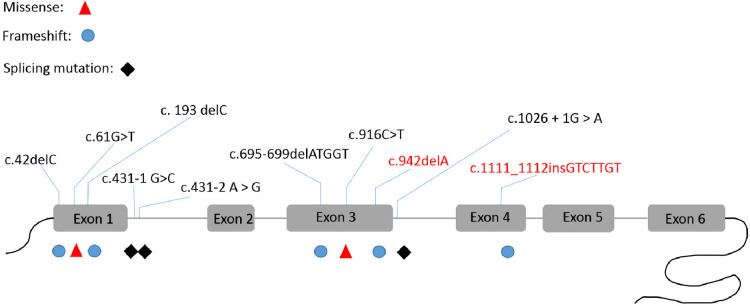
The *SLC16A2* pathogenic variants that were reported in China. This is the first report of c.942delA and c.1111_1112insGTCTTGT frame-shift variants in this gene.

Patient 2 was a boy who came to our hospital at 6 months of age due to motor retardation. A physical examination revealed his height (66.2 cm) and weight (8.2 kg) were normal. He had neck muscle weakness, limb hypertonia, head retroflexion, microcephaly (39.5 cm, <−2 SD), epilepsy, muscle hyperirritability and poor motor coordination. T2-weighted MRI imaging (T2WI) revealed a low signal intensity in the left inferior and middle cerebellar peduncle that was not obvious in the posterior limbs of the internal capsules (Figures 1E,E1–E3). Thyroid function tests revealed abnormal levels of T3 (5.78 nmol/L, normal range 1.32–4.07 nmol/L), T4 (53.32 nmol/L, normal range 73–206 nmol/L), free T3 (15.72 nmol/L, normal range 3.3–8.95 nmol/L) and free T4 (6.73 nmol/L, normal range 11.9–25.6 nmol/L). His mother's thyroid function test results suggested a decrease in T4: 3.49 (normal range 5.00–13.00) pg/dl). The proband's elder brother had similar but more severe symptoms (Figures 1C,C-1–C–4). He presented with microcephaly, epilepsy, profound intellectual disability and severe cerebral palsy. His thyroid function was also abnormal (T3: 2.78 nmol/L, T4: 4.39 ug/dl, free T3: 9.23 pmol/L and free T4:12.02 pmol/L). A brain CT scan at the age of 4 months reported no significant abnormality (Figure 1 for characteristics of the patients).

### Genetic testing

We identified a hemizygous variant c.942delA (Val315fs*28) of *SLC16A2* (NM_006517.4, ChrX:73,744,559 in exon 3) in patient 1 and c.1111_1112insGTCTTGT (Gly375fs*6) (NM_006517.4, ChrX:73,745,669 in exon 4) in patient 2 by using WES.

Sanger sequencing revealed that the c.942delA (Val315fs*28) and c.1111_1112ins GTCTTGT (Gly375fs*6) variants were inherited from the mother of patients 1 and 2, respectively (Figure 2). Four *in silico* tools were used to predict the impact of the two novel variants (Figure 3B). Predictive software suggested that c.942delA (Val315fs*28) and c.1111_1112insGTCTTGT (Gly375fs*6) were harmful variants. The Swiss-Model software was used to predict the three-dimensional structure of the wild-type (WT) and variant of the *SLC16A2* proteins. By comparison, it was clear that variants had altered the protein length and overall shape of the *SLC16A2* protein. Variants can cause a frameshift which results in premature truncation of the protein that is likely to cause major changes in protein activity. The protein products are usually more unstable and are easily degraded. This is demonstrated diagrammatically in Figures 3A,A-1,A-2. Thus, both variants can be classified as pathogenic with supporting evidence such as PVS1 + PM2-supporting + PP4 + PP1. According to the Orphanet (www.orphadata.org), at least 132 families with 320 affected individuals had been reported worldwide. We searched the databases (OMIM, Wanfang, CNKI and PubMed) for reported Chinese AHDS patients. To date only 9 families with AHDS have been reported in China. An extensive literature review was conducted (Table 1).

**Table 1:** Clinical characteristics of 11 patients with AHDS syndrome in China.

	P1^18^	P2^18^	P3^18^	P4^18^	P5^18^	P6^19^	P7^20^	P8^21^	P9^22^	Proband 1	Proband 2	Ration in China	Ration in literature^23^
										This report			
Age	8.75 year	0.75 year	0.75 year	1.25 year	0.75 year	0.67 year	1 year	1 year	0.33 year	0.08 year	0.5 year	1.44 ± 1.33	∼0.65 year
Sex	M	M	M	M	M	M	M	M	M	M	M	100% M	100% M
Full-term infant	+	+	+	+	+	36 ^+ 3^W	+	+	+	+	+	90.91%	99.3%
Family history	–	–	–	–	–	–	–	NA	–	–	–	0%	0%
Low Weight (kg)	17.2 (<−3SD)	7.6 (−1SD∼−2SD)	9 (Normal)	9.5 (−1SD)	8 (−1SD∼−2SD)	8.8 (−1SD)	7 (−2SD)	NA	5.6 (−1SD∼−2SD)	3.04 kg (<−3SD)	8.2 kg (Normal)	80.00%	37%–66.6%
Abnormal head circumferences (cm)	NA	NA	NA	NA	NA	44 cm (−1SD)	45 cm (−2SD)	NA	NA	33 cm (<−2SD)	39.5 cm (<−2SD)	100.00%	10%–33.3%
Motor delay	+	+	+	+	+	+	+	+	+	+	+	100.00%	100%
Psychomotor retardation	+	+	+	+	+	+	+	+	+	+	+	100.00%	100%
Speech	No speech	No speech	No speech	No speech	No speech	No speech	No speech	NA	No speech	No speech	No speech	100.00%	19.9%–69%
Muscular hypotonia	+	+	+	+	+	+	+	+	+	+	+	100.00%	74%–100%
Muscle hyperirritability	+	+	+	+	+	+	+	+	+	+	+	100.00%	70.8%–94%
Babinski's sign	+	+	+	+	+	–	NA	NA	NA	–	+	75.00%	NA
Abnormal Cranial MRI result	Demyelination (8year, 9 months)	–	–	Demyelination (5 m)	Demyelination (7 m)	cerebra gap widened, Delayed myelination	delayed myelination and mild cortical atrophy	local changes in the white matter around the posterior corners of the bilateral ventricles	cerebra gap widened (bilateral frontoparietal); Delayed myelination (anterior limb of internal capsule); Pachygyria (bifrontal lobes)	–	a patchy long T1 and long T2 signal shadow in the left mid-cerebellar peduncle, with a slightly high signal on high b-value DWI and a slightly low signal on ADC.	72.73%	33.1%–79.1%
Sinus tachycardia	+	+	+	+	+	NA	NA	NA	+	+	+	100.00%	0%–1.4%
Thyroid hormone levels	TT3: (–)	TT3: (–)	TT3: (↑)	TT3: (↑)	TT3: (↑)	TT3: (↑)	TT3: NA	TT3: NA	TT3: NA	TT3: (–)	TT3: (↑)	90.91%	27.9%–66.6%
	FT3: (↑)	FT3: (↑)	FT3: (↑)	FT3: (↑)	FT3: (↑)	FT3: NA	FT3: (↑)	FT3: (↑)	FT3: (↑)	FT3: (–)	FT3: (↑)		
	TT4: (↓)	TT4: (↓)	TT4: (↓)	TT4: (↓)	TT4: (↓)	TT4: (↓)	TT4: NA	TT4: (↑)	TT4: NA	TT4: (–)	TT4: (↓)		
	FT4: (↓)	FT4: (↓)	FT4: (↓)	FT4: (↓)	FT4: (↓)	FT4: NA	FT4: (↓)	FT4: (↑)	FT4: (↓)	FT4: (–)	FT4: (↓)		
	TSH: (–)	TSH: (–)	TSH: (–)	TSH: (–)	TSH: (–)	TSH: (–)	TSH: (–)	TSH: NA	TSH: (–)	TSH: (–)	TSH: (–)		
Maternal Thyroid Function	–	–	NA	NA	–	–	–	NA	+	+	+	37.50%	70.83%
Others	Long face, Narrow forehead, Open mouth, Thick vermilion border, Tented upper lip vermilion	–	–	Long face, Narrow forehead, Open mouth, Thick vermilion border, Tented upper lip vermilion	–	Long face; Cupped ear; Esotropia; Pneumonia; Spasticity; Athetosis; Scoliosis; Cryptorchidism; Dyspnea; Hyperbilirubinemia; G6PD	elongated face with bifrontal narrowing and flat nose	NA	Pneumonia; Malnutrition	Pneumonia; Lacticaciduria; Conjunctival hyperemia; Vomiting; Poor appetite; Laryngeal stridor Malnutrition; apathy; low immunoglobulin and iron levels	Seizures Pneumonia Long face; Cupped ear; Esotropia	90.91%	31%–75%
Variation types	missense	missense	missense	Frameshift	Frameshift	Splicing mutation	Splicing mutation	Splicing mutation	Frameshift	Frameshift	Frameshift	NA	NA
Variations	c.916C > T (p. Q306*)	c.916C > T (p. Q306*)	c.61G > T (p. E21*)	c.695-699delATGGT (p. N232Sfs*7)	c.42delC, (p. W15Gfs*69)	c.431-1 G > C	c.431-2 A > G	c.1026 + 1G > A	c. 193 del C (p. P65Rfs*19)	c.942del A(p. V315fs*28)	c.1111_1112insGTCTTGT(p. G375fs*6)	NA	NA
Genetic origin	Mo (Mother)	Mo	De novo	De novo	Mo	Mo	Mo	De novo	Mo	Mo	Mo	De:Mo = 1:2.67	De:Mo = 1:2.43

## Discussion

AHDS is a disorder of brain development that leads to moderate to severe intellectual disability and problems with movement. The *SLC16A2* gene is the only known causal gene. The gene is widely expressed in tissues and it may play a key role in the development of the central nervous system (9, 10). In males, loss-of-function variants in this gene are associated with psychomotor retardation, but in females there is no neurological deficit, and the thyroid-deficiency phenotype is mild (11). Pathogenic variants in *SLC16A2* have been found in diverse populations around the world (12–14).

Two novel variants are described in this study, c.1111_1112insGTCTTGT (Gly375fs*6) and c.942delA (Val315fs*28). In addition, we summarized all the reported mutants that cause AHDS syndrome in Chinese patients (Figure 4). The variants reported in this study are all frameshift variants. Both resulted in premature stop codons that could act as nonsense-mediated mRNA decay (PVS1). Furthermore, neither were found to be present in controls, the 1,000 Genome Project and the Exome Sequencing Project (PM2-supporting). Rare SNPs have very broad and deleterious effects on phenotypes when compared to the weak effects of common SNPs (15). The clinical presentation of all the affected individuals in these families are consistent with the symptoms of AHDS (PP4), and the genotype co-segregated with the phenotype in at least one family tested (PP1). Thus both novel variants can be classified as pathogenic.

The clinical presentations of AHDS includes neurologic findings, pyramidal and extrapyramidal findings, seizures and dysthyroidism (16, 17). There are considerable inter- and intra-familial differences in phenotypes. In severe cases, the patient will be unable to walk or speak. Our two patients exhibited many of these clinical features, including hypotonia, feeding difficulties, dystonia and choreoathetosis. We have compiled the clinical phenotypes, laboratory tests results and molecular characteristics of the 9 cases previously reported in China (Table 1) (18–22). We compared the clinical characteristics of Chinese patients with those reported in the literature and found that the most common clinical manifestations are psychomotor retardation (Chinese cohort: literature, 100:100%), motor delay (Chinese cohort: literature, 100:100%) muscular hypotonia (Chinese cohort: literature, 100:74%–100%) and muscle hyperirritability (Chinese cohort: literature, 100:70.8%–94%). The above symptoms are the core clinical symptoms of AHDS syndrome. Other symptoms include low weight (Chinese cohort: literature, 80.0:37–66.6%), microcephaly (Chinese cohort: literature, 100:10–33.3%). sinus tachycardia (Chinese cohort: literature, 100.0:1.4), abnormal thyroid function (Chinese cohort: literature, 90.91:27.9–66.6%) and abnormal brain MRIs (Chinese cohort: literature, 72.73:33.1–79.1%). Although reported in several patients (Table 1, P2–3), delayed myelination is not a consistent feature necessarily associated with neurological symptoms of AHDS.

We should also consider the possibility of AHDS in males having typical thyroid and neurological symptoms. Phenotypic severity is generally thought to be related to the residual transport function of the MCT8 protein. Some pathogenic missense variants and in-frame SNP deletions are associated with milder clinical phenotypes (6). Some patients die early on from recurrent infections and/or aspiration pneumonia (23). Currently, only one case of a female patient has been reported, the rest being males. Most heterozygous women have no neurological symptoms, but thyroid function tests may be mildly abnormal, possibly due to altered X chromosome inactivation (24).

In this study, the percentage of abnormal maternal thyroid function was only 37.50%. Some researchers believe that a thyroid hormone level with a free T3/T4 ratio above 0.75 is helpful in diagnosing patients. However, in this study, only 67% (6/9) of patients fulfilled this condition (24). It can be seen that the method has limited diagnostic value in detecting AHDS. By itself, it is not sufficient as a diagnostic indicator but it can guide the molecular diagnosis. All variants reported in China have been assessed as pathogenic or likely pathogenic variants according to the ACMG guidelines. Among these, the variants were mainly inherited from the mother, and the *de novo* rate is 27.27%.

At present, there is no report of incomplete penetrance of AHDS, but there was heterogeneity in presentation of clinical severity. However, mutations in the *SLC16A2* gene can lead to differences in MTC8 transport function and thus differences in neurological symptoms in the patients (7, 25). Our patients presented with similar facial features, such as a long face, thick and tented upper lip, open mouth, of which these may have been due to prenatal and infantile hypotonia. In addition, the patients in this study also exhibited some heterogeneous clinical presentation clinical symptoms, including lacticaciduria, hyperkalemia, conjunctival hyperemia and laryngeal stridor. Low immunoglobulin and iron levels and vomiting are also potential novel clinical features of the disease. These symptoms may be related to the novel variants, or they may be non-specific. Due to the young age of the patients in this study, some neurological symptoms may yet manifest themselves. The lacticaciduria and low immunoglobulin levels seen may be associated with infection and malnutrition, but this will need to be confirmed by further studies involving more cases. Unfortunately, due financial difficulties, the family was unwilling to be proceed with further tests and examinations. However, we will continue to follow-up these families in an effort to further improve the relevant data regarding AHDS.

The heterogeneous manifestations of AHDS make it difficult for clinicians to diagnose the disease based on the clinical presentation alone. Other disorders with X-linked or autosomal recessive inheritance patterns such as MECP2 duplication syndrome, X-linked progressive cerebellar ataxia, Pelizaeus-Merzbacher disease and non-goitrous congenital hypothyroidism 6 could exhibit overlapping features. These include hypotonia, progressive spasticity, absent speech, hypomyelination, abnormal thyroid hormones and severe intellectual disability and they are all candidates for differential diagnosis.

AHDS is a rare disorder of brain development. The presentation of abnormal relative concentrations of three circulating thyronine molecules along with severe neurological abnormalities should provide diagnostic clues of AHDS. WES is a valuable test for identifying the causal genes in these patients. According to the literature, the majority of patients were diagnosed with AHDS when there were either clinical symptoms combined with WES or WES alone. Currently, there is no effective treatment for AHDS. Observational studies have shown that 3,3′,5-triiodothyroacetic acid (TRIAC) can restore some thyroid functions, and thus provides a possible treatment option (26).

## Conclusions

In this study, two novel pathogenic variants in the *SLC16A2* gene were identified in two Chinese patients who exhibited symptoms of neurodevelopmental delay, extrapyramidal (dystonia, chorea and tardive dyskinesia), and pyramidal (spasticity). The variants described in this study expand the phenotypic spectrum of patients with different ethnic backgrounds, which are valuable for the genetic diagnosis and future variant-based screening for AHDS.

## Data Availability

The datasets presented in this study can be found in online repositories. The names of the repository/repositories and accession number(s) can be found in the article/**Supplementary Material**.

## References

[1] Masnada S Sarret C Antonello CE Fadilah A Krude H Mura E Movement disorders in MCT8 deficiency/Allan-Herndon-Dudley syndrome. Mol Genet Metab. (2022) 135:109–13. 10.1016/j.ymgme.2021.12.003 34969638

[2] Dumitrescu AM Liao XH Best TB Brockmann K Refetoff S. A novel syndrome combining thyroid and neurological abnormalities is associated with mutations in a monocarboxylate transporter gene. Am J Hum Genet. (2004) 74:168–75. 10.1086/380999 14661163 PMC1181904

[3] Friesema EC Grueters A Biebermann H Krude H von Moers A Reeser M Association between mutations in a thyroid hormone transporter and severe X-linked psychomotor retardation. Lancet. (2004) 364:1435–7. 10.1016/S0140-6736(04)17226-7 15488219

[4] van Geest FS Gunhanlar N Groeneweg S Visser WE. Monocarboxylate transporter 8 deficiency: from pathophysiological understanding to therapy development. Front Endocrinol. (2021) 12:723750. 10.3389/fendo.2021.723750 PMC8440930 34539576

[5] Vancamp P Demeneix BA Remaud S. Monocarboxylate transporter 8 deficiency: delayed or permanent hypomyelination? Front Endocrinol. (2020) 11:283. 10.3389/fendo.2020.00283 PMC7237703 32477268

[6] Masnada S Groenweg S Saletti V Chiapparini L Castellotti B Salsano E Novel mutations in SLC16A2 associated with a less severe phenotype of MCT8 deficiency. Metab Brain Dis. (2019) 34:1565–75. 10.1007/s11011-019-00464-7 31332729

[7] Friesema EC Jansen J Heuer H Trajkovic M Bauer K Visser TJ. Mechanisms of disease: psychomotor retardation and high T3 levels caused by mutations in monocarboxylate transporter 8. Nat Clin Pract Endocrinol Metab. (2006) 2:512–23. 10.1038/ncpendmet0262 16957765

[8] Li Q Wang K. Intervar: clinical interpretation of genetic variants by the 2015 ACMG-AMP guidelines. Am J Hum Genet. (2017) 100:267–80. 10.1016/j.ajhg.2017.01.004 28132688 PMC5294755

[9] Vatine GD Shelest O Barriga BK Ofan R Rabinski T Mattis VB Oligodendrocyte progenitor cell maturation is dependent on dual function of MCT8 in the transport of thyroid hormone across brain barriers and the plasma membrane. Glia. (2021) 69:2146–59. 10.1002/glia.24014 33956384

[10] Felmlee MA Jones RS Rodriguez-Cruz V Follman KE Morris ME. Monocarboxylate transporters (SLC16): function, regulation, and role in health and disease. Pharmacol Rev. (2020) 72:466–85. 10.1124/pr.119.018762 32144120 PMC7062045

[11] Dumitrescu AM Liao XH Weiss RE Millen K Refetoff S. Tissue-specific thyroid hormone deprivation and excess in monocarboxylate transporter (mct) 8-deficient mice. Endocrinology. (2006) 147:4036–43. 10.1210/en.2006-0390 16709608

[12] Novara F Groeneweg S Freri E Estienne M Reho P Matricardi S Clinical and molecular characteristics of SLC16A2 (MCT8) mutations in three families with the Allan-Herndon-Dudley syndrome. Hum Mutat. (2017) 38:260–4. 10.1002/humu.23140 27805744

[13] Islam MS Namba N Ohata Y Fujiwara M Nakano C Takeyari S Functional analysis of monocarboxylate transporter 8 mutations in Japanese Allan-Herndon-Dudley syndrome patients. Endocr J. (2019) 66:19–29. 10.1507/endocrj.EJ18-0251 30369548

[14] Grijota-Martinez C Barez-Lopez S Gomez-Andres D Guadano-Ferraz A. MCT8 Deficiency: the road to therapies for a rare disease. Front Neurosci. (2020) 14:380. 10.3389/fnins.2020.00380 32410949 PMC7198743

[15] Bomba L Walter K Soranzo N. The impact of rare and low-frequency genetic variants in common disease. Genome Biol. (2017) 18:77. 10.1186/s13059-017-1212-4 28449691 PMC5408830

[16] Beheshti R Aprile J Lee C. Allan-Herndon-Dudley syndrome: a novel pathogenic variant of the SLC16A2 gene. Cureus. (2022) 14:e21771. 10.7759/cureus.21771 35251841 PMC8890594

[17] Noorian S Hamzehlou S Rabbani A Sotoudeh A Pour Rostami K Savad S. The role of thyroid function tests in diagnosing Allan-Herndon-Dudley syndrome revisited: a novel Iran-based mutation. Basic Clin Neurosci. (2021) 12:563–8. 10.32598/bcn.2021.1924.1 35154596 PMC8817177

[18] Wang J Zhang Q Bao X Chen Y Yu S. Clinical and genetic features of five patients with Allan-Herndon-Dudley syndrome. Zhonghua Yi Xue Yi Chuan Xue Za Zhi. (2018) 35:484–8. 10.3760/cma.j.issn.1003-9406.2018.04.005 30098239

[19] Tang YL Peng J Xiong J Pang N Wu LW Yang HY A family with Allan-Herndon-Dudley syndrome due to SLC16A2 gene mutation. Zhonghua Er Ke Za Zhi. (2018) 56:829–34. 10.3760/cma.j.issn.0578-1310.2018.11.008 30392207

[20] Chen X Liu L Zeng C. A novel variant in SLC16A2 associated with typical Allan-Herndon-Dudley syndrome: a case report. BMC Pediatr. (2022) 22:180. 10.1186/s12887-022-03259-5 35382784 PMC8981932

[21] Wang A Xi J Yuan F Wang Y Wang S Wang C Generation of an induced pluripotent stem cell line (SHCDNi003-A) from a one-year-old Chinese Han infant with Allan-Herndon-Dudley syndrome. Stem Cell Res. (2020) 46:101872. 10.1016/j.scr.2020.101872 32603881

[22] Qianfang ZF Qingyang CUI. Allan-Herndon-Dudley syndrome caused by a novel mutation of SLC16A2 gene: a case report and literature review. J Clin Pediatr. (2020) 38:953–6. 10.3969/j.issn.1000-3606.2020.12.017

[23] Remerand G Boespflug-Tanguy O Tonduti D Touraine R Rodriguez D Curie A Expanding the phenotypic spectrum of Allan-Herndon-Dudley syndrome in patients with SLC16A2 mutations. Dev Med Child Neurol. (2019) 61:1439–47. 10.1111/dmcn.14332 31410843

[24] Quesada-Espinosa JF Garzon-Lorenzo L Lezana-Rosales JM Gomez-Rodriguez MJ Sanchez-Calvin MT Palma-Milla C First female with Allan-Herndon-Dudley syndrome and partial deletion of X-inactivation center. Neurogenetics. (2021) 22:343–6. 10.1007/s10048-021-00660-7 34296368

[25] Armour CM Kersseboom S Yoon G Visser TJ. Further insights into the Allan-Herndon-Dudley syndrome: clinical and functional characterization of a novel MCT8 mutation. PLoS One. (2015) 10(10):e0139343. 10.1371/journal.pone.0139343 26426690 PMC4591285

[26] Kersseboom S Horn S Visser WE Chen J Friesema EC Vaurs-Barriere C In vitro and mouse studies supporting therapeutic utility of triiodothyroacetic acid in MCT8 deficiency. Mol Endocrinol. (2014) 28:1961–70. 10.1210/me.2014-1135 25389909 PMC5414784

